# Treatment of femoral neck fractures using actis stem: complication rate in 188 uncemented hemiarthroplasties

**DOI:** 10.1007/s00402-024-05352-z

**Published:** 2024-05-09

**Authors:** L. Leitner, F. Schitz, P. Sadoghi, P. Puchwein, J. Holinka, A. Leithner, E. Kalcher

**Affiliations:** 1 Department of Orthopedics and Trauma, Medical University of Graz, Graz, Austria; 2 Department of Orthopaedics and Trauma Surgery, Musculoskeletal University Center Munich (MUM), LMU University Hospital, Munich, Germany; 3 Department of Orthopedics and Trauma Surgery, Hanusch-Krankenhaus, Vienna, Austria

**Keywords:** Femoral neck fracture, Actis, Hemiarthroplasty, Uncemented

## Abstract

**Introduction:**

Cemented hemiarthroplasty (HA) is preferred in treating dislocated femoral neck fractures in elderly, osteoporotic patients, since uncemented HA was associated with mechanical complications more frequently. Cementation can conversely cause cardiopulmonary complications, leading to demand on safe, uncemented implants addressing osteoporosis. This study is set up as a retrospective feasibility study on the use of an uncemented, collared wedge implant (Actis®, DePuy Synthes, Warsaw, IN), for HA in elderly patients, focusing on complication rate.

**Materials and methods:**

From 1,194 patients, treated with HA in two study centers between 2017–2022, 188 received Actis® uncemented stem with bipolar head. Complete follow-up were retrospectively collected in all patients.

**Results:**

In 188 patients (f: 64.9%; age: 83.1 ± 7.7a) included, no case of intra-operative mortality was recorded. 2 day mortality was 1.1%, 30 day mortality was 7.4% and 1 year mortality was 28.2%. 2 (1.1%) intra-operative fractures did not receive surgical revision, 3 (1.6%) post-operative periprosthetic fractures caused separate admission and revision. 2 cases (1.1%) of early infection required surgical revision.

**Conclusion:**

Our data provide proof of concept, that Actis® Stem allows an alternative, uncemented treatment option for displaced femoral neck fractures with HA. In case of preoperative or intraoperative medial cortical bone defects, stability of this implant is deteriorated.

**Supplementary Information:**

The online version contains supplementary material available at 10.1007/s00402-024-05352-z.

## Introduction

Due to global population ageing, growing elderly demographic, and increasing life expectancy, it is projected that the annual incidence of displaced femoral neck fractures might increase up to 21.3 million by the year 2030 [12, 18]. Considering these growth numbers and anticipated health care expenses in the near future, the ability of effective treatment of these patients, whilst minimizing complication rate, seems critical [7].

Hemiarthroplasty (HA) is a widespread surgical technique for the treatment of displaced intracapsular femoral neck fractures in frail patients. Whilst traditionally, cemented implantation is mostly preferred in elderly patients, studies showed promising results using uncemented fixation more recently [9]. Although uncemented fixation is correlated with an increased number of mechanical complications [13], it has been increasingly adopted (1) to avoid cement-associated cardiopulmonary complications, (2) due to a marked improvement in the reliability of fixation (even in highly osteoporotic bone, often represented by a Dorr type B and C femur) and (3) because of a shorter operation time [2, 21]. Cementation of HA is still discussed controversially, since a recent systematic review by Elmenshawy et al. in 1561 bipolar HAs revealed significantly lower blood loss (p < 0.0001), shorter operative time (p < 0.0001), less infection (p = 0.03) and also a lower risk of heterotopic ossification (p = 0.007) for uncemented HAs, whilst on the other hand patients with cemented HA suffered from significantly less pain (p < 0.0001) [9].

Especially in old, highly morbid patients, with highest risk for cement-associated cardiopulmonary complications [23]. As several well-known risk factors for bone cement implantation syndrome (BCIS) are known [25], especially in medically complex patients with impaired cardiopulmonary function anesthesiologists at our study centers therefor encourage utilization of uncemented techniques.

The use of an uncemented, collared wedge implant (Actis®, DePuy Synthes, Warsaw, IN) became popular at our Trauma Centers, driven by good experiences in the reliability of fixation due to common use in primary total hip arthroplasty. Actis® stem is a worldwide commonly used, uncemented, collared, wedge implant for primary THA. Especially the collar, which has been added to this implant, offers a new field of discussion in the context of uncemented HA, since recent data from the German Arthroplasty Registry (Endoprothesenregister Deutschland (EPRD)) also suggest that a collared stem design reduces the risk of periprosthetic femoral fractures in uncemented total hip arthroplasty [16]. The discussion and analysis of these implant features remains especially relevant in the femoral neck fracture cohort, since undiagnosed and untreated osteoporosis remains a main issue with this diagnosis [20].

Since no published data exist for Actis® use in hip fracture surgery to our knowledge, this study aimed to establish the mortality and revision surgery outcome data for a representative series of HA, using this uncemented implant. By comparing our results to earlier published data on cemented HA, we wanted to evaluate whether this technique remains a reasonable treatment option despite contradictory recommendations, or whether we should switch to a cemented system.

## Methods

### Ethics statement

This study was approved by the local Institutional Ethical Review Board (Reference number: EK-Nr. 34–281 ex 21/22). All experiments were performed in accordance with relevant guidelines and regulations; informed consent was obtained from all participants.

### Study population

Between January 2017 and September 2022, 1,194 patients treated with femoral neck fracture in our two study centers (Medical University of Graz, Styria, Austria and Hanusch-Krankenhaus, Vienna, Austria), were retrieved from our data system. From these cases, 188 patients (15.7%) treated with HA with an uncemented, collared wedge implant (Actis®, DePuy Synthes,) were identified, and therefore retrospectively included in this study. Surgery was performed via anterolateral or direct anterior approach depending on the study center. Main criteria for the use of this implant for this indication were mainly (1) surgeons’ preference and/or (2) statement from anesthesiologists, concerning severe patients’ risk factors for BCIS, most commonly in medically complex patients with impaired cardiopulmonary function [25]. Inclusion criteria was the treatment of traumatic femoral neck fracture using Actis® stem between June 2017 and June 2022, exclusion criteria was the use of another uncemented stem design or the use of cemented technique (using fourth-generation cementation technique [10]).

### Data collection

Case history and clinical follow up were retrieved from the hospital intern and associated centers data systems using keyword identification of all written examination reports, mortality data/dates were received from insurance data. Medical history (date of implantation, infection, revision surgeries, clinical follow-ups; identified germ(s), concomitant diseases at surgery; last clinical follow-up examination) demographic characteristics (age, sex), radiologic follow-up, and a current, complete follow-up on survival were collected from all patients. The medical follow-up data (e.g. outpatients’ department visits) were retrieved from our hospital database systems, also including data from other regional public hospitals (minimizing the chance of undetected revision surgeries in other public hospitals). Dorr classification was assessed according to the original publication by Dorr LD et al. [8].

### Statistical methods

SPSS Statistics 20 (IBM, Armonk, NY) was used for data analysis. Data were expressed as frequencies or percentages for discrete variables and as mean ± standard deviation for continuous variables. Comparisons between groups were made using the chi-square test for categorical variables and the Student t-test for continuous variables, when normally distributed. The Kolmogorov–Smirnov test was used for assessment of normal distribution. A two sided p-value < 0.05 was considered to be statistically significant.

## Results

**Fig. 1:**
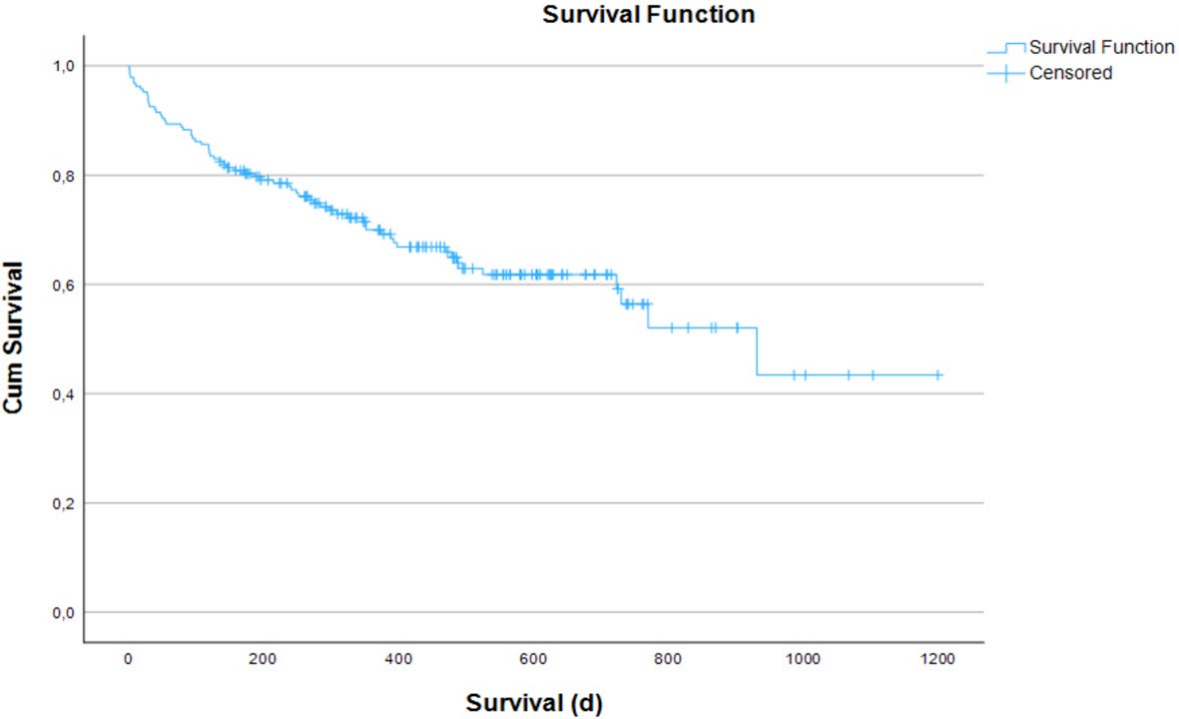
Kaplan Meier survival analysis of all included patients, showing comparable mortality rate to earlier published data [24]

In 188 (f: n = 122, 64.9%); age: 83.1 ± 7.7a) patients treated with an uncemented, collared wedge implant for femoral neck fracture (Actis®), no case of intra-operative mortality was recorded (Fig. 1). Basic demographic data of our study cohort is displayed in (Table 1). 48 h post-OP mortality was 1.1% (2/188; 98a, cardiac arrest; 59a, preexistent chronic thromboembolic pulmonary hypertension (CTEPH) in a palliative setting), 30-day post-OP mortality 7.4% (14/188), 1 year mortality was 28.2% (53/188) and 35,1% (66/188) deceased during complete follow-up (Fig. 1).

**Table 1:** Baseline characteristics of included patients

Age (years)	
N	188
Mean	83,1
Range	59 – 99
Std. deviation	7,7
Gender	
N	188
Female	122 (64,9%)
Male	66 (35,1%)
Follow-Up (days)	
N	188
Mean	107,7
Range	0 – 980
Std. deviation	153,6
Outpatient follow-up (days)	
N	141
Mean	142
Range	14 – 980
Std. deviation	163,6
Time from surgery to death (days)	
N	66
Mean	212
Range	0 – 930
Std. deviation	209
Time from surgery to revision (days)	
N	5
Mean	29
Range	10 – 35
Std. deviation	17,3
Cause of revision	
Infection	N = 2
Periprosthetic fracture	N = 3
with luxation N = 2	
Mortality post-OP	
48 h	N = 2 (1,1%)
30 day	N = 14 (7,4%)
1 year	N = 53 (28,2%)
during follow-up	N = 66 (53,1%)

**Fig. 2:**
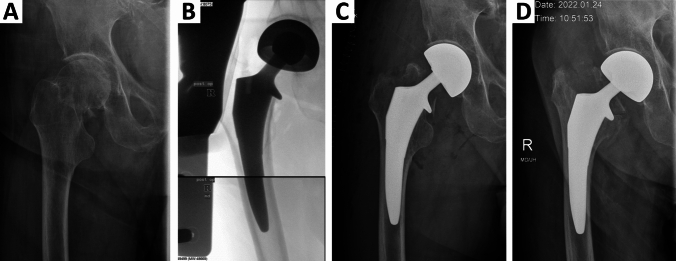
(**a**) X-ray showing a case of femoral neck fracture in a 96-year-old man. Although the operation-reports describes a fracture of the medial cortical bone region, (**b**) intraoperative fluorescence reveals successful implantation. (**c**) X-ray performed 2 days after surgery due to ongoing pain, revealing a medial cortical bone and major trochanter tip fracture. Due to reduced patient´s overall health state and preoperative immobility of the patient, surgical revision was not indicated. (**d**) Two months after surgery X-ray reveals further subsidence, but a clinically stable implant situation

**Fig. 3:**
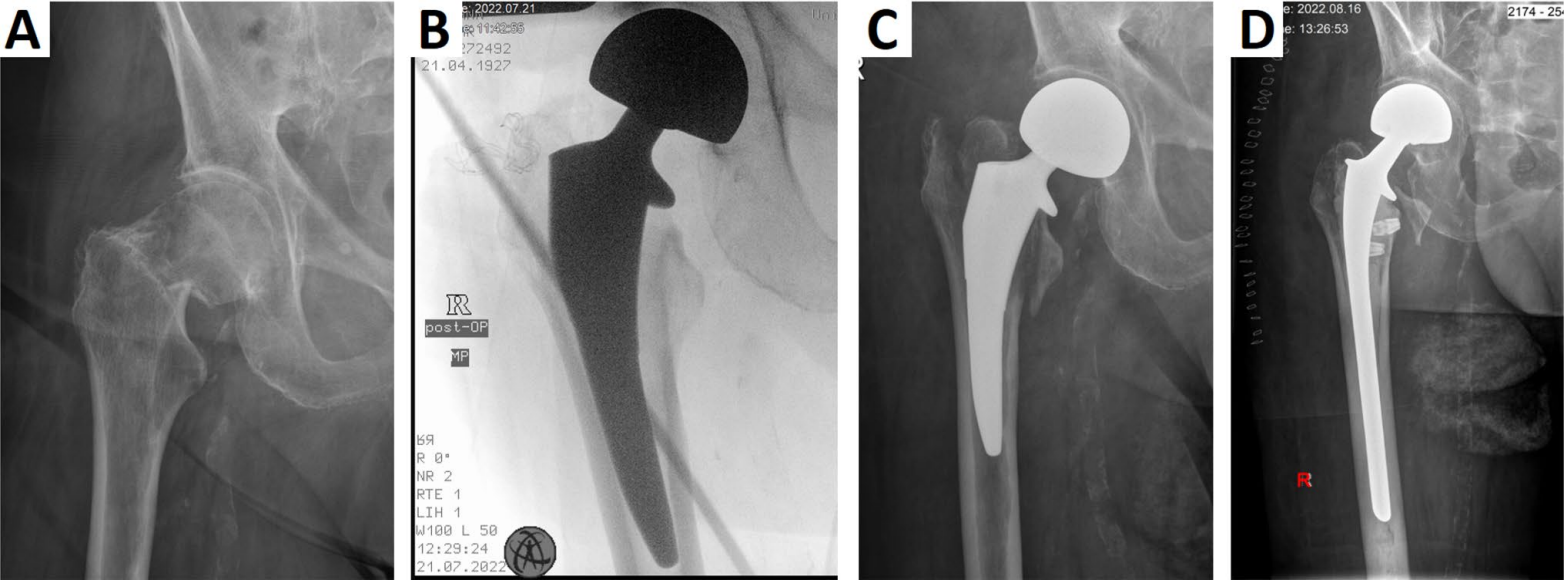
(**a**) X-rays showing a case of femoral neck fracture in a 95-year-old man. An additional medial cortical bone defect can be observed in intraoperative luminescence, (**b**) probably caused by excessive osteotomy. (A rather secondary, but definite reason for a weak calcar) (**c**) X-ray due to occurring pain during mobilization 10 days after surgery revealed a fracture of the major and minor trochanter region, leading to instable implant. (**d**) Revision was performed using a cemented long stem, allowing immediate mobilization of this frail patient

An overall number of 5 (2.7%) periprosthetic fractures were observed during a mean follow-up of 385,9 days. Two (1.1%) intra-operative fractures occurred but did not demand or receive intra- or postoperative surgical revision (Fig. 2). In 3 cases (1.6%) post-operative periprosthetic fractures caused separate admission and revision surgery after 2, 3 and 5 months, of which two fractures, involving the trochanter region (Fig. 3), were treated with cemented stems and one was treated with open reduction and internal fixation. Mean age of all mechanical complication cases was 88.0 ± 8.9a, and therefore not statistically significant older than the rest of the cases (p = 0.12). Four of these cases had a Dorr-C, one a Dorr-B type femur. Interestingly, a pre- or post-OP defect or deficiency of the medial cortical bone/minor trochanter region can be described in all 5 cases where fractures occurred. In detail, the medial cortex and trochanter minor were fractured in both cases with intra-OP fractures, whilst in all 3 post-OP fracture cases, the collar could not be placed on the calcar region due to pre-existing or intraoperatively described defect of the collar region.

All involved surgeons in these five cases with mechanical complications had a case number lower than 40 hip arthroplasties per year.

Two cases (1.1%) of early infection occurred within 10 and 24 days, and were successfully treated with debridement, antibiotics and implant retention (DAIR).

No case of implant loosening occurred during the observational period. We found no significant difference in complication rate between using direct anterior approach (DAA) and anterolateral approach (Watson-Jones).

## Discussion

Cemented HA is considered the gold standard treatment for displaced femoral neck fractures in elderly patients [13]. This is supported by recent evidence from registry data, cemented HA appears to be associated with lower frequency of mechanical complications and reoperations, also revealing similar mortality data for cemented and uncemented HA [29]. This represents a controversial outcome, since traditionally, cement pressurization is considered to increase fat embolization and fatal cement implantation syndrome, especially in patients with severe comorbidities due to increased intramedullary pressure [9, 19]. In this context, it must be mentioned that registry data always contain a certain selection bias [28], e.g. medically complex, highly frail patients with impaired cardiopulmonary function and increased mortality could have been treated in the uncemented group more frequently, offering a possible explanation for this controversial outcome from registry data. Especially in patients with recently described risk factors for severe BCIS (i.e. age > 75, American Society of Anesthesiologists Class III or IV, and renal failure) [25], anesthesiologists at our study centers often encourage utilization of uncemented techniques. Our data on Actis® stem offer an uncemented fixation opportunity in these cases.

Furthermore, recent studies suggest that femur morphology, which is usually not displayed in registry data, is a main predictor for periprosthetic fractures [4]. Additionally, shorter operative time [21], the avoidance of cement-associated cardiopulmonary complications[25], and marked improvement on the reliability of fixation in modern implants, which are not depicted in registry data as yet, might justify the further evaluation of uncemented HA. This consideration might also have led to the recommendation to rather compare individual implants than broad categories of implants in a recent publication of the New Zealand Registry study group [5].

Finally, surgeons’ prevalence to a certain fixation type (another factor difficult to identify retrospectively), can influence the selected implant. This aspect has earlier been described, surgeons with a strong preference for fixation type are less likely to deviate from their preferred method of fixation based on the characteristics of the patient before them [22].

Due to all of these factors, a certain number of patients will more likely receive uncemented HA due to surgeon’s intraoperative decision, often based on the anesthesiologists’ recommendation to not use cement fixation [25], as this was the case in 15.7% of our patient cohort. Although a selection bias towards patients in worse medical conditions in our uncemented cohort could be assumed, our patients had comparable one year post-operative survival rates to earlier published data on cemented and uncemented HA [24].

**Fig. 4:**
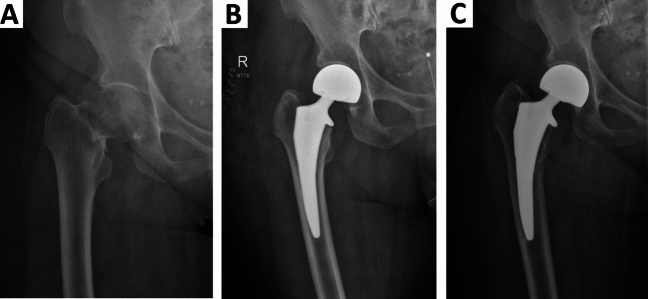
(**a**) X-rays showing a case of femoral neck fracture in an 88-year-old woman, (**b**) treated with Actis® stem HA 1 week and (**c**) 2,7 years following surgery. Successful, complication-free clinical follow-up and radiological osteointegration was observed in this case. HA, Hemiarthroplasty

Due to good experience with Actis® stem in THA, it was also frequently used in HA cases in our study centers, where cemented fixation had to be avoided as described above. This is the first published series of displaced femoral neck fractures treated with Actis® stem HA in 188 cases, revealing promising results (Fig. 4).

In accordance with earlier published data, suggesting improved fixation capability of Actis® stem compared to Corail® (DePuy Synthes, Warsaw, IN) stem, our data for the first time suggests that this can also be seen in the treatment of femoral neck fractures [15]. From our perspective, it is an easy to use stem with highly improved reliability of fixation, even in Dorr type B and C femur types, more often seen in elderly patients [7]. High reliability of fixation is considered particularly important in this group, since osteoporosis is often undiagnosed and untreated in cases with femoral neck fractures [20].

Aseptic reoperation rate in our study was 2.7%, which is lower than earlier published data on a comparable tapered titanium wedge implant with metaphyseal hydroxyapatite (Taperloc®, Biomet, Indiana, USA) [21]. The most prominent additional feature of the Actis® stem compared to the Taperloc® is the collar. There is increasing evidence from infinite element analysis, that a collar might improve primary stability of stem fixation [14, 17]. This is supported by recent publications, that a collar may particularly ensure sufficient torsional stability of a stem during the phase of primary stability, before osteointegration [3, 6]. Recent data from the German Arthroplasty Registry (Endoprothesenregister Deutschland (EPRD)) also suggest that a collared stem design reduces the risk of periprosthetic femoral fractures [16]. This might have been especially relevant in our study population, mainly suffering from severe osteoporosis. Although actual evidence from prospective randomized trials suggests modest but significant lower risk of periprosthetic fracture rates in cemented compared to uncemented group [11, 24], the promising results of modern collared stem designs might further reduce this gap [27].

Since Actis® is a metaphyseal fitting stem, fracture of the medial cortical bone region has earlier been described as critical event during implantation [15]. Due to the loss of stability of the osseous ring in the metaphyseal region, and the absence of stability provided by the collar, the stress on the remaining bone anchoring might be reduced. This is in line with findings from our study, where a preexisting or intraoperative defect of the medial cortical bone region could be observed in all cases with mechanical failure (Fig. 2, 3). In both cases this mechanically relevant defect of the metaphyseal region led to impairment of the main principle of fixation and stability of the implant, additionally affecting the stabilizing effect of the collar.

According to the authors understanding, these cases of mechanical failure could have been avoided, if the surgeons had switched to cemented fixation in this situation. Based on this finding we recommend cementation, or diaphyseal anchoring stem designs if cementation should be avoided.

All cases with mechanical complications were carried out by surgeons with a case number lower than 40 primary arthroplasties in the previous year. Especially in cases with reduced bone quality, it seems crucial to have certain knowledge on implant capability and judgmental skills concerning method change if necessary (e.g. cementation in case of medial cortical bone defect). This is in line with earlier findings, indicating an annual surgeon volume < 35 cases is associated with increased risk of complications in hip arthroplasty [26], and has also been shown in HA [1]. In this context it should be mentioned that other authors concluded that experienced arthroplasty surgeons with significant expertise in press-fitting techniques may achieve similar outcomes to cemented femoral stem fixation [30].

## Study limitations

A limitation of this study is that it was mainly aimed as a retrospective proof of concept for the safe treatment of intracapsular femoral neck fractures using uncemented Actis® stem, which has not earlier been published. Therefore, further conclusions to be drawn from this study are extremely limited and there is nearly no generalization possible.

Another limitation of this study is, that the retrospective design mainly allowed analyzation of the radiologic outcome (e.g. periprosthetic fractures, loosening) and implementation/inclusion of patient reported outcome measures (PROMs) and functional outcome was not possible. Evaluation of PROMs for this treatment remains relevant, since a recent meta-analysis demonstrated significantly less postoperative thigh pain in cemented HAs [9]. Due to the retrospective design of this study, osteoporosis was often undetected during acute transmission to our surgical department, reflecting a common clinical situation [20]. Although treatment is always indicated in our discharge letters, we could not include this diagnosis in our statistical analysis.

Due to the retrospective design of this study, a certain rate of revision surgeries lost to follow up can occur. We have not solely checked the medical records from our study centers, but also had access to information from other public hospitals in our area, so we consider the rate of revision surgeries lost to follow up to be very low.

Although this study presents an initial proof of concept in this field, the authors believe that further prospective studies, based on our data, remain necessary from this point to address these limitations.

## Conclusion

Our data provide proof of concept, that the Actis® stem allows safe treatment for displaced femoral neck fractures with HA, which we consider a safe treatment method, especially when cementation has to be avoided. Caution should be taken in case of preoperative or intraoperative defects of the medial cortical bone of the femoral neck, which was correlated to all cases of mechanical implant failure in our series – and therefor represents a relative contraindication for this treatment in our opinion. For detection of these defects, careful preoperative radiological assessment and sufficient intraoperative judgmental skills, often based on surgeons’ experience, are crucial. In such cases, we recommend a switchover to cemented technique, or diaphyseal anchoring stem designs if cementation should be avoided. Surgeons who choose to use Actis® stem for this indication should be encouraged to be familiar with the implant specific fixation principles and contraindications, since complications seem to be correlated with low surgeons’ case number according to our results.

Further prospective studies will be needed to further assess PROMs and functional outcome using this technique. Further research and follow-up studies are also warranted to establish the long-term outcomes and durability of this treatment option.

### Supplementary Information

Below is the link to the electronic supplementary material.

Supplementary file1 (PDF 199 KB)

## Data Availability

Due to privacy and ethical restrictions, the data that support the findings of this study is not available publicly. However, a summary of the data and any additional information required for replication of the study can be provided upon request from the corresponding author. In compliance with the journal's data sharing policy, we are committed to making the data underlying this research available to interested researchers in a timely and transparent manner. Access to the data will be granted after a formal request is submitted and is subject to approval by the institutional review board (IRB) or ethics committee, ensuring that all privacy and ethical considerations are met. We believe in the importance of transparency and reproducibility in research, and we are dedicated to facilitating access to our data to promote further scientific inquiry and collaboration.
